# History of Incarceration and Its Association With Geriatric and Chronic Health Outcomes in Older Adulthood

**DOI:** 10.1001/jamanetworkopen.2022.49785

**Published:** 2023-01-06

**Authors:** Ilana R. Garcia-Grossman, Irena Cenzer, Michael A. Steinman, Brie A. Williams

**Affiliations:** 1 Department of Medicine, University of California, San Francisco; 2 National Clinician Scholars Program, Philip R. Lee Institute of Health Policy Studies, University of California, San Francisco; 3 San Francisco VA Medical Center, San Francisco, California; 4 Division of Geriatrics, University of California, San Francisco; 5 Center for Vulnerable Populations, University of California, San Francisco

## Abstract

**Importance:**

Although incarcerated older adults experience higher rates of chronic disease and geriatric syndromes, it is unknown whether community-dwelling older adults with a history of incarceration are also at risk for worse health outcomes.

**Objective:**

To evaluate the association between a history of incarceration and health outcomes, including chronic health conditions and geriatric syndromes, in older age.

**Design, Setting, and Participants:**

This cross-sectional study using population-based data from the nationally representative Health and Retirement Study included US community-dwelling adults aged 50 years or older who completed the 2012 or 2014 survey waves assessing self-reported history of incarceration. Statistical analysis was completed from December 2021 to July 2022.

**Exposures:**

Self-reported history of incarceration.

**Main Outcomes and Measures:**

Geriatric health outcomes included cognitive impairment, mobility impairment, vision impairment, hearing impairment, urinary incontinence, and impairment of activities of daily living (ADLs). Chronic health outcomes included high blood pressure, diabetes, chronic lung disease, heart disease, stroke, mental health conditions, heavy alcohol use, and self-reported health. Survey weights were applied to adjust for the survey design.

**Results:**

Among 13 462 participants, 946 (7.6%) had experienced incarceration (mean [SD] age, 62.4 [7.8] years); compared with 12 516 people with no prior incarceration (mean [SD] age, 66.7 [10.0] years), previously incarcerated adults were more likely to be male (83.0% vs 42.8%; *P* < .001) and in the lowest quartile of wealth (44.1% vs 21.4%; overall *P* < .001). After adjusting for age, sex, race and ethnicity, wealth, educational attainment, and uninsured status, a history of incarceration was associated with a 20% to 80% increased risk of all geriatric syndromes evaluated, including impairment of ADLs (relative risk [RR], 1.62; 95% CI, 1.40-1.88) and hearing impairment (RR, 1.22; 95% CI, 1.04-1.44). Incarceration was also associated with increased risk of some chronic diseases, including chronic lung disease (RR, 1.56; 95% CI, 1.27-1.91), mental health conditions (RR, 1.80; 95% CI, 1.55-2.08), and heavy alcohol use (RR, 2.13; 95% CI, 1.59-2.84). Prior incarceration was not associated with diabetes or cardiovascular conditions.

**Conclusions and Relevance:**

In this study, at least 1 in 15 older US adults reported a history of incarceration in their lifetime. Past incarceration was associated with many chronic diseases and geriatric syndromes even after accounting for socioeconomic status. These findings suggest that attention to incarceration history may be an important consideration in understanding and mitigating health risks in older age.

## Key Points

**Question:**

Is a history of incarceration associated with chronic diseases or geriatric syndromes in older community-dwelling adults?

**Findings:**

In this nationally representative cross-sectional study of 13 462 community-dwelling US adults aged 50 years or older, 7.6% of participants had experienced incarceration. After controlling for socioeconomic status, a history of incarceration was associated with increased risk of several geriatric syndromes and chronic health conditions in older age.

**Meaning:**

These findings suggest that clinicians and policy makers should consider targeted interventions to mitigate the health impacts of incarceration among older community-dwelling adults.

## Teaser

This cross-sectional study of data from the Health and Retirement Study examines the association of history of incarceration with risk of chronic health conditions and geriatric syndromes among older community-dwelling US adults.

## Introduction

Carceral populations in the US grew dramatically between 1980 and 2020, increasing approximately 400% in jails and 800% in prisons.^1,2,3^ At the same time, the proportion of incarcerated older adults also rose precipitously.^4^ As a result, many community-dwelling older adults have a lifetime history of incarceration since nearly 95% of incarcerated people are eventually released to the community.^5^ However, little is known about the health impacts of incarceration in later life or even the precise number of people in the US who have experienced incarceration during their lifetime.^6^ This is in part due to the fragmentation of data among county, state, and federal correctional institutions and the limited number of national population-based surveys that ask participants about their history of incarceration.^7,8^

Incarcerated older adults have higher rates of chronic medical conditions compared with their nonincarcerated peers.^9,10^ In addition, incarcerated older adults experience higher rates of geriatric syndromes at earlier ages, including urinary incontinence, hearing impairment, and activity of daily living (ADL) impairments (eg, difficulty toileting, bathing, dressing, and feeding oneself).^11^ This evidence of accelerated aging has led both correctional institutions and researchers to consider incarcerated adults to be older or geriatric at the age of 50 years.^4,12^ The underlying mechanisms leading to higher rates of disease in this population have been poorly studied; possible etiologies include exposure to acute and chronic stress, variable access to high-quality health care, and incarceration’s association with other social determinants of health, including homelessness, unemployment, and food insecurity.^4,11,13,14^ Although studies^9,11^ have examined the associations between incarceration and poor health among older adults who are incarcerated, it is unknown how many community-dwelling older adults have experienced incarceration or whether a history of incarceration confers risk for worse health outcomes in older adults.

Understanding the association between incarceration and health among community-dwelling older adults has important clinical and policy implications. Such knowledge could inform the need for health care professionals to screen patients for a history of incarceration and could support the development of interventions to minimize the long-term effects of incarceration after release. Additionally, policy makers and public health advocates are increasingly advocating for alternatives to incarceration; establishing data on population incarceration rates and on the longitudinal impacts of incarceration is important for evaluating the impacts of alternatives to incarceration. Therefore, we examined whether a history of incarceration was associated with higher rates of chronic diseases and geriatric syndromes in a nationally representative sample of community-dwelling US adults aged 50 years or older.

## Methods

### Study Design and Population

We performed a cross-sectional study of data from the 2012 and 2014 survey waves of the Health and Retirement Study (HRS). The HRS is a nationally representative longitudinal survey of adults aged 50 years or older that is conducted by the University of Michigan and sponsored by the National Institute on Aging.^15^ The HRS enrolls participants every 6 years using a multistage sampling design to create a representative cohort of community-dwelling older US adults.^15,16,17^ Institutionalized persons, including those residing in carceral facilities, are excluded from enrollment in the HRS. Participants complete interviews every 2 years about their economic, health, and psychosocial information. Our study included all participants who completed the 2012 or 2014 psychosocial leave-behind questionnaire, the only survey waves that assessed self-reported incarceration history. The questionnaire was left with participants to return by mail and was administered to a random 50% of all HRS respondents in 2012 and the other 50% in 2014.^18^ Approval for this data analysis was obtained from the institutional review board at the University of California, San Francisco, which waived the patient consent requirement because the data are deidentified. We followed the Strengthening the Reporting of Observational Studies in Epidemiology (STROBE) reporting guideline.

### Measures

#### Incarceration

Our exposure variable was self-reported incarceration, defined as ever having spent time in juvenile detention, jail, or prison. Participants reported whether the total time spent incarcerated was less than 1 month, less than 1 year, between 1 and 5 years, more than 5 years, or unknown. In some analyses, we dichotomized incarceration length (<1 month vs ≥1 month) due to small sample sizes.

#### Sociodemographic Characteristics

Sociodemographic characteristics included self-reported age, sex, race and ethnicity (Black/African American, Hispanic/Latino[a], White, and other [Asian, American Indian, Alaska Native, Native Hawaiian, and other Pacific Islander]), educational attainment, veteran status, and a history of ever experiencing homelessness. All variables were drawn from the survey year corresponding to the completed questionnaire.

We assessed socioeconomic status using wealth (an assessment of all assets and debts) since it is the most comprehensive measurement of socioeconomic status in older adults.^19^ We categorized wealth into 4 quartiles from lowest (quartile 1) to highest (quartile 4).

#### Health Outcomes

The HRS assesses self-reported physical health outcomes, which have been well validated in older US adults.^20^ Chronic health conditions included ever having been told by a physician that you have a health condition, including high blood pressure or hypertension, diabetes or high blood glucose level, chronic lung disease, stroke, and heart disease (myocardial infarction, coronary heart disease, angina, congestive heart problems, or other heart problems). A history of mental health conditions was defined as being told by a physician that you have any emotional, nervous, or psychiatric problem. We categorized self-reported heavy alcohol use as drinking more than 4 alcoholic drinks daily. We categorized self-rated health as excellent, very good, or good vs fair or poor.

The HRS also collected information about geriatric conditions. Cognitive impairment was defined as ever being told by a physician that you have Alzheimer disease, dementia, senility, or serious memory impairment. We defined mobility impairment as using an assistive device (cane, walker, or wheelchair) or difficulty walking several blocks. Vision and hearing impairment were defined as reporting fair or poor vision or hearing. Urinary incontinence was defined as having experienced loss of control of urine in the past 12 months. Impairment in ADLs was defined as having difficulty with bathing, dressing, feeding, toileting, or transferring; impairment in instrumental ADLs (IADLs) was defined as having difficulty with meal preparation, grocery shopping, taking medications, making telephone calls, or managing money.

### Statistical Analysis

Data were analyzed from December 2021 to July 2022. We conducted a descriptive analysis of sociodemographic factors, health care utilization, and health outcomes stratified by history of incarceration. To compare individuals with and without a history of incarceration, we used a Rao-Scott χ^2^ test for categorical variables and a nonparametric test for equality of medians for continuous variables. We applied survey weights to adjust for the survey design.

To compare the prevalence of health outcomes among those with and without a history of incarceration, we used modified Poisson regression and controlled for age, sex, race and ethnicity, wealth quartiles, educational attainment, and uninsured status. To better understand the association of biological vs social factors with health outcomes, we also performed a minimally adjusted analysis, adjusting for age and sex. In a combined analysis, we evaluated the risk of having any geriatric syndrome and any chronic health condition. We identified potential confounders a priori based on existing literature and controlled for variables that might confound the variable-outcome association. We did not adjust for variables that are also potential mediators between history of incarceration and adverse health outcomes, including homelessness, substance use, or mental illness. We performed regression analyses using a complete case analysis given the low rates of missingness for included variables (<0.4%).

Next, we assessed the impact of length of incarceration. Participants who were incarcerated for an unknown duration (n = 30) were excluded from this analysis. We used descriptive statistics and a trend analysis to compare health outcomes among participants with no incarceration history, those who had been incarcerated for less than 1 month, and those who had been incarcerated for 1 month or more. To evaluate whether there was a difference in health outcomes among those with shorter vs longer periods of incarceration, we repeated the modified Poisson regression, controlling for the same variables, and compared individuals with 1- to 30-day incarceration durations vs 1 month or more. We used a 2-sided a priori significance threshold of *P* < .05. We completed our analyses using Stata, version 17.0 (StataCorp LLC) and SAS, version 9.4 (SAS Institute, Inc).

## Results

In this nationally representative sample of 13 462 adults aged 50 years or older, 946 (7.6%) reported being incarcerated during their lifetime, representing 4 625 413 US adults. Compared with 12 516 people with no history of incarceration (92.6% of the sample, representing 63 427 605 US adults), participants with any incarceration history were younger (mean [SD] age, 62.4 [7.8] years vs 66.7 [10.0] years; *P* < .001) and more likely to be male (83.0% vs 42.8%; *P* < .001), Black/African American (18.8% vs 9.1%) or Hispanic/Latino(a) (12.3% vs 8.1%) (overall *P* < .001), and in the lowest quartile of wealth (44.1% vs 21.4%; overall *P* < .001) (Table 1). Participants with a history of incarceration had a median total wealth of $43 000 (IQR, $1500-$195 000) compared with $204 000 (IQR $47 000-$566 000) among those who had never been incarcerated (*P* < .001). Previously incarcerated participants were more likely to report having General Educational Development or not graduating from high school (31.9% vs 16.8%; overall *P* < .001), to have ever experienced homelessness (27.7% vs 3.1%; *P* < .001), and to be a veteran (31.7% vs 16.7%; *P* < .001).

**Table 1.** Participant Characteristics and Health Outcomes Stratified by History of Incarceration

Characteristic	History of incarceration (N = 13 462)^a^		*P* value
	No (n = 12 516)^b^	Yes (n = 946)^c^	
Age, y			
50-59	2738 (28.9)	383 (46.2)	<.001
60-69	3727 (36.8)	323 (36.5)	
70-79	3919 (21.2)	194 (13.9)	
≥80	2132 (13.1)	46 (3.5)	
Sex			
Female	7739 (57.2)	202 (17.0)	<.001
Male	4777 (42.8)	744 (83.0)	
Race and ethnicity			
Black/African American	1900 (9.1)	283 (18.8)	<.001
Hispanic/Latino(a)	1358 (8.1)	132 (12.3)	
White	8896 (79.6)	497 (65.0)	
Other^d^	362 (3.2)	34 (3.9)	
GED or less than high school education	2493 (16.8)	328 (31.9)	<.001
Wealth			
Median (IQR), $	204 000 (47 000-566 000)	43 000 (1500-195 000)	<.001
Quartile^e^			
1	2937 (21.4)	447 (44.1)	<.001
2	3094 (23.6)	253 (27.7)	
3	3223 (26.2)	143 (16.4)	
4	3262 (28.8)	103 (11.9)	
Veteran	2149 (16.7)	298 (31.7)	<.001
Ever homeless	403 (3.1)	263 (27.7)	<.001
Health care utilization			
Usual place of care			
None	1446 (11.0)	154 (16.7)	<.001
Emergency department or other	496 (3.7)	98 (9.9)	
Primary care	10 508 (85.2)	689 (73.1)	
Visits to physician in past 2 y, median (IQR), No.	6 (3-12)	5 (2-11)	.17
Overnight hospital stay in past 2 y	3021 (22.5)	276 (26.4)	.04
Uninsured	760 (6.0)	151 (17.0)	<.001
Insurance type			
Medicaid	1000 (7.0)	181 (15.5)	<.001
Medicare	7987 (53.6)	437 (39.6)	<.001
Veterans Affairs	734 (5.4)	68 (7.2)	.14
Private	6976 (62.4)	360 (42.4)	<.001
≥1 Type	3704 (25.9)	123 (11.9)	<.001

Abbreviation: GED, General Educational Development.

^a^ Data are presented as absolute number (weighted percentage) of participants unless otherwise indicated. As this was a population-based sample, we estimated prevalence for the overall US population.

^b^ Represents 63 427 605 adults.

^c^ Represents 4 625 413 adults.

^d^ Includes Asian, American Indian, Alaska Native, Native Hawaiian, and other Pacific Islander.

^e^ Wealth was categorized into 4 quartiles from lowest wealth (quartile 1) to highest wealth (quartile 4).

### Incarceration History

Among those who had ever been incarcerated (n = 946), the majority were incarcerated for less than 1 month (60.9%). One-fifth (20.5%) were incarcerated for 1 to 12 months, 11.7% for 1 to 5 years, and 4.0% for more than 5 years.

### Health Outcomes

After adjusting for age, sex, race and ethnicity, wealth, educational attainment, and uninsured status, a history of incarceration was associated with a 20% to 80% increased risk of each geriatric syndrome (Table 2). These included cognitive impairment (relative risk [RR], 1.80; 95% CI, 1.09-2.97; *P* = .02), mobility impairment (RR, 1.31; 95% CI, 1.18-1.46; *P* < .001), urinary incontinence (RR, 1.46; 95% CI, 1.26-1.69; *P* < .001), vision impairment (RR, 1.26; 95% CI, 1.12-1.42; *P* < .001), hearing impairment (RR, 1.22; 95% CI, 1.04-1.44; *P* = .02), ADL impairment (RR, 1.62; 95% CI, 1.40-1.88; *P* < .001), and IADL impairment (RR, 1.55; 95% CI, 1.32-1.81; *P* < .001). Incarceration history was also associated with an increased risk of some chronic health conditions, including chronic lung disease (RR, 1.56; 95% CI, 1.27-1.91; *P* < .001), mental health conditions (RR, 1.80; 95% CI, 1.55-2.08; *P* < .001), and heavy alcohol use (RR, 2.13; 95% 1.59-2.84; *P* < .001), in addition to fair or poor self-rated health (RR, 1.22; 95% CI, 1.09-1.37; *P* = .001) (Table 2). Prior incarceration was not significantly associated with high blood pressure, diabetes, heart disease, or stroke. When we adjusted for only age and sex, risk ratios were higher for all chronic health conditions and geriatric syndromes than in our fully adjusted model (Table 2). In a combined analysis, participants with a history of incarceration were more likely to have any geriatric syndrome (69.0% vs 58.5%; *P* < .001) and any chronic health condition (85.0% vs 75.1%; *P* < .001). When controlling for the same aforementioned factors, an incarceration history was associated with increased risk of any geriatric syndrome (RR, 1.20; 95% CI, 1.12-1.28; *P* < .001) and any chronic health condition (RR, 1.09; 95% CI, 1.04-1.13; *P* < .001).

**Table 2.** Health Outcomes Associated With History of Incarceration Among Older Adults

Outcome	Participants, No. (%) (N = 13 462)^a^		RR (95% CI)		*P* value
	No prior incarceration (n = 12 516)	Prior incarceration (n = 946)	Adjusted for age and sex	Fully adjusted^b^	
Geriatric syndromes					
Cognitive impairment	269 (2.2)	27 (3.2)	2.51 (1.50-4.19)	1.80 (1.09-2.97)	.02
Mobility impairment	3998 (28.6)	367 (36.2)	1.72 (1.55-1.90)	1.31 (1.18-1.46)	<.001
Vision impairment	2842 (20.7)	322 (32.4)	1.79 (1.56-2.05)	1.26 (1.12-1.42)	<.001
Hearing impairment	2602 (19.5)	262 (28.6)	1.45 (1.25-1.67)	1.22 (1.04-1.44)	.02
Urinary incontinence in past year	3238 (24.6)	198 (21.6)	1.49 (1.28-1.73)	1.46 (1.26-1.69)	<.001
ADL impairment^c^	2090 (15.6)	247 (27.0)	2.29 (1.95-2.68)	1.62 (1.40-1.88)	<.001
IADL impairment^d^	1727 (13.3)	205 (22.4)	2.29 (1.97-2.66)	1.55 (1.32-1.81)	<.001
Chronic health conditions					
Fair or poor self-rated health	3253 (24.0)	370 (37.9)	1.76 (1.55-1.98)	1.22 (1.09-1.37)	.001
High blood pressure	7668 (56.2)	602 (59.1)	1.13 (1.05-1.21)	1.01 (0.94-1.08)	.76
Diabetes	2947 (21.5)	262 (24.6)	1.17 (1.03-1.33)	0.97 (0.85-1.11)	.66
Chronic lung disease	1203 (8.9)	155 (15.1)	2.12 (1.76-2.56)	1.56 (1.27-1.91)	<.001
Heart disease^e^	3145 (22.8)	239 (23.1)	1.12 (0.98-1.28)	1.05 (0.91-1.21)	.50
Stroke	1032 (7.3)	88 (8.5)	1.39 (1.04-1.87)	1.13 (0.84-1.52)	.40
Mental health condition	2115 (18.0)	266 (30.5)	2.05 (1.79-2.35)	1.80 (1.55-2.08)	<.001
Heavy alcohol use^f^	211 (2.1)	85 (10.7)	2.51 (1.86-3.40)	2.13 (1.59-2.84)	<.001

Abbreviations: ADL, activity of daily living; IADL, instrumental activity of daily living; RR, relative risk.

^a^ Absolute numbers with weighted percentages are shown.

^b^ Adjusted for age, sex, race and ethnicity, wealth, educational attainment, and uninsured status.

^c^ Difficulty with bathing, dressing, feeding, toileting, or transferring.

^d^ Difficulty with meal preparation, grocery shopping, taking medications, making telephone calls, or managing money.

^e^ Includes history of myocardial infarction, coronary heart disease, angina, or congestive heart failure.

^f^ Defined as more than 4 drinks per day.

### Incarceration Length

Despite small absolute numbers, all health outcomes that were associated with a history of incarceration except cognitive impairment also had a statistically significant test of trend across length of incarceration, suggesting that longer incarceration periods may be associated with an increased risk of these health outcomes (Figure). However, in our adjusted Poisson regression model, there were no significant differences between individuals who were incarcerated for 1 month or more vs less than 1 month (eTable in Supplement 1).

**Figure.**
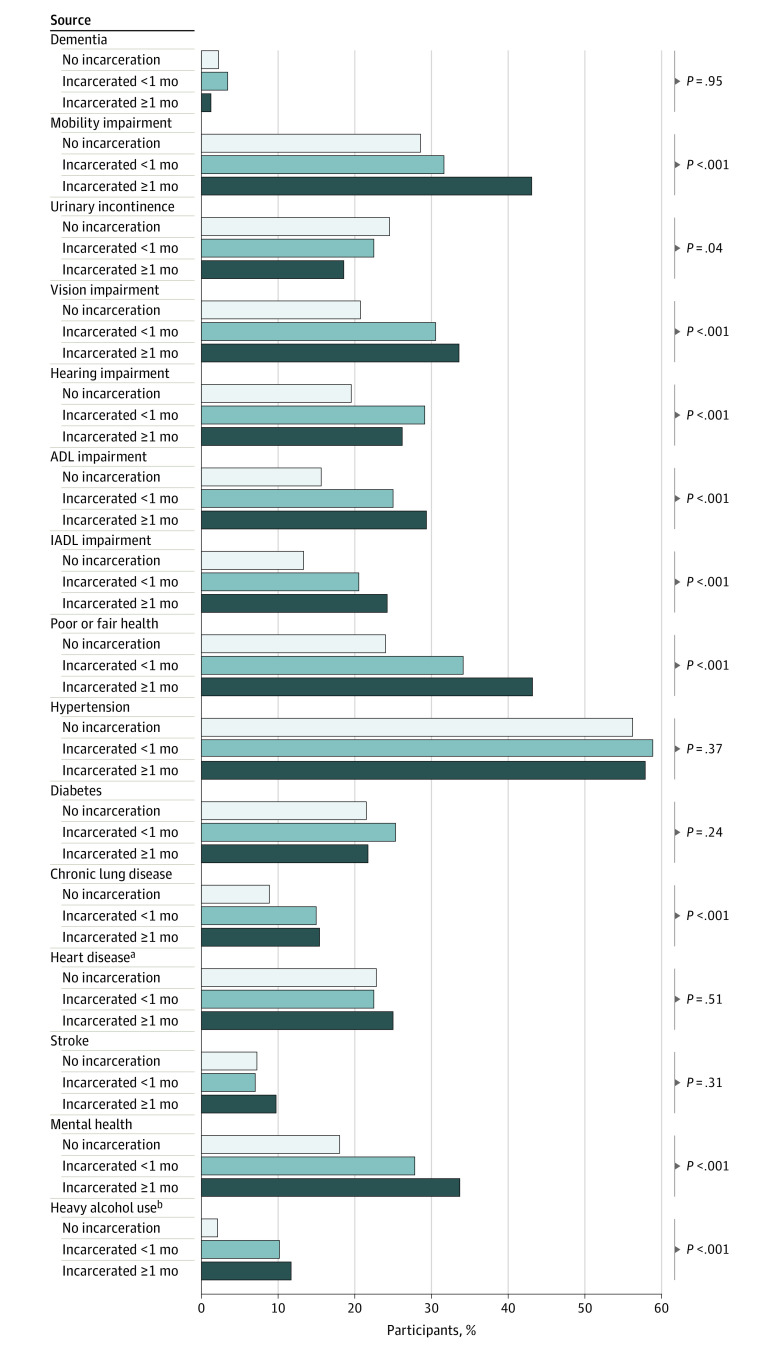
Prevalence of Geriatric Syndromes and Chronic Health Conditions, Stratified by History and Length of Incarceration *P* values represent test of trend across all 3 incarceration levels (none, <1 month, or ≥1 month). ADL indicates activity of daily living; IADL, instrumental activity of daily living. ^a^Includes history of myocardial infarction, coronary heart disease, angina, or congestive heart failure. ^b^Defined as more than 4 drinks per day.

## Discussion

In a nationally representative sample of older US adults, we found that at least 1 in 15 adults (7.6%) aged 50 years or older had experienced incarceration during their lifetime. This is likely an underestimate of the actual experience of older US adults since the HRS does not include people who were unhoused, did not have a telephone, or were incarcerated at the time of study enrollment. As there is a profound lack of data about US adults with a criminal legal history, this study is among the first to estimate self-reported history of incarceration among community-dwelling older US adults. There is a critical need to improve data collection and transparency to generate accurate estimates of lifetime incarceration in the US.^7,8,21^ Nevertheless, our findings indicate that incarceration is so prevalent in the US that an older adult’s likelihood of having a history of incarceration is higher than their lifetime risk of developing colorectal cancer.^22^ Yet, despite the ubiquity of criminal legal system involvement in the US, there has been relatively little funding and research dedicated to understanding the downstream health outcomes and needs of this population.^21,23^

To our knowledge, this is the first published study to demonstrate that a lifetime history of incarceration was associated with increased risk of geriatric syndromes and several chronic health conditions among community-dwelling older adults. We found that older adults with any incarceration history (even of short duration) had a 20% to 80% increased risk of experiencing geriatric syndromes even after controlling for socioeconomic factors. These findings are consistent with prior research demonstrating a high prevalence of chronic health conditions and geriatric syndromes at relatively young ages among older adults who were currently incarcerated (rather than living in the community)^10,11^ and support the notion of accelerated aging in this population.

Our results regarding chronic health outcomes were variable. People who had been incarcerated had an increased risk of fair or poor self-rated health in addition to health outcomes related to substance use or mental health concerns, including chronic lung disease, mental health conditions, and heavy alcohol use. Incarceration history was not associated with cardiovascular outcomes (high blood pressure, heart disease, or stroke) or diabetes. Prior research about the increased prevalence of these conditions among incarcerated populations is mixed. Age-adjusted rates of hypertension are higher among incarcerated people compared with the general population^11,24^; however, these studies were unable to account for possible confounders. One study^9^ found that after adjusting for sociodemographic factors and alcohol consumption, incarcerated adults of all ages had higher odds of being diagnosed with hypertension but similar rates of diabetes and myocardial infarction compared with nonincarcerated adults.

We found that health outcomes associated with a history of incarceration had a positive linear trend in our unadjusted analysis; longer incarceration periods were associated with higher disease prevalence for many health outcomes (Figure). However, we found no statistically significant differences between individuals who had been incarcerated for 1 month or more vs less than 1 month after adjusting for socioeconomic covariates. However, this analysis was limited by statistical power; most reported incarceration events were short, and only 15% of participants were incarcerated for longer than 1 year. This suggests that a larger longitudinal cohort study that observes adults after incarceration is warranted to better understand the dose-response relationship between exposure to incarceration and adverse health outcomes.

This study was not designed to assess the underlying causal pathways between incarceration and poor health, although many potential explanations exist. It is possible that people who experience incarceration have worse baseline health before they enter jail or prison that persists into older age following incarceration. Additionally, exposure to incarceration may exacerbate poor health outcomes through exposure to trauma and violence, acute and chronic stress from living in dehumanizing conditions, and/or variable access to healthy food, physical activity, and high-quality health care. It is also possible that incarceration leads to downstream barriers to other social determinants of health, such as employment or housing, which in turn contribute to poor health.^25,26,27^ Finally, it is possible that the associations between incarceration and poor health outcomes are the result of unaccounted confounders, suggesting the need for a longitudinal cohort study of people following release from incarceration. Regardless of the underlying etiology, our results support that a history of incarceration is an important marker for risk of poor health outcomes and thus should be considered by clinicians, public health professionals, and policy makers.

Overall, this study suggests that having a history of incarceration is an important factor associated with poor health in older age. Furthermore, it suggests that both health care professionals and policy makers should consider the health implications of incarceration both for older adults who were recently released from jail or prison and for older patients with an incarceration history, even if that history was remote. To further elucidate the health impacts of incarceration over the life course, population-based health studies should collect detailed information about criminal legal involvement.^7,8^ Future research should explore the temporal relationships between incarceration events and health outcomes over the life course, the role of recurrent incarceration events, and the effects of long-term incarceration on the health and well-being of older adults. Given that incarceration is differentially experienced by racial and ethnic minority individuals in the US, there is a need for additional research to evaluate whether involvement in the criminal legal system contributes to health disparities seen in these populations. From a clinical perspective, this study suggests that it is time to invest in research that evaluates the utility and impact of screening for incarceration in primary care settings as a part of standard social-risk screening. Furthermore, health care professionals may benefit from enhanced training, for example, about the effects of incarceration on individuals and communities or the existence and implications of accelerated aging in this population. Public health and health care professionals should also prioritize implementation and evaluation of interventions that are designed to mitigate the long-term poor health outcomes present among people who have experienced incarceration to determine whether they can ameliorate these health disparities in older age. Evidence about the long-term consequences of incarceration on the health of communities is critical as policy makers increasingly consider ways to reduce the footprint of mass incarceration in the US.

### Limitations

Several limitations should be considered when interpreting the results of our study. Data were not available about the number or timing of each participant’s incarceration event(s). Most participants who had experienced incarceration reported relatively short periods of detainment. As a result, our ability to study the impact of incarceration length was limited and our results may not be generalizable to people who experience long periods of incarceration. The HRS has minimal data on housing, substance use, and mental health, limiting our ability to assess the role of medical and social comorbidities in the association between incarceration and health outcomes and again underscoring the critical need for high-quality longitudinal data that would allow for such analyses.

## Conclusions

In this cross-sectional study of a nationally representative sample of community-dwelling older US adults, at least 1 in 15 had a history of incarceration. Past incarceration was associated with many geriatric syndromes and chronic diseases even after accounting for socioeconomic status. These findings suggest that attention to incarceration history may be valuable for understanding and mitigating health risks in older age.

## Notes

Supplement 1. **eTable.** Health Outcomes Among Older Adults With Longer Incarcerations Compared With Shorter Incarcerations

Supplement 2.
Data Sharing Statement
