# Supplementary material for: History of Incarceration and Its Association With Geriatric and Chronic Health Outcomes in Older Adulthood
Source: JAMA Netw Open. 2023 Jan 6;6(1):e2249785. doi: 10.1001/jamanetworkopen.2022.49785 (PMC9856648; doi:10.1001/jamanetworkopen.2022.49785)
Supplement: Supplement 1. — eTable. Health Outcomes Among Older Adults With Longer Incarcerations Compared With Shorter Incarcerations [file jamanetwopen-e2249785-s001.pdf]

Supplementary Online Content

Garcia-Grossman IR, Cenzer I, Steinman MA, Williams BA. History of incarceration and its association with geriatric and chronic health outcomes in older adulthood. *JAMA Netw Open*. 2023;6(1):e2249785. doi:10.1001/jamanetworkopen.2022.49785

eTable. Health Outcomes Among Older Adults With Longer Incarcerations Compared With Shorter Incarcerations

This supplementary material has been provided by the authors to give readers additional information about their work.

© 2023 Garcia-Grossman IR et al. *JAMA Network Open*.

eTable. Health Outcomes Among Older Adults With Longer Incarcerations (≥1 mo) Compared With Shorter Incarcerations (<1 mo)

Participants, No. (%) (N = 916[a])[b]				
	**Outcome**	**<1 month incarceration (n = 553)**	**≥1 month incarceration (n = 363)**	**Fully adjusted[c] RR (95% CI), p-value**
Geriatric syndromes	Cognitive impairment	14 (3.4)	8 (1.3)	Sample size too small
	Mobility impairment	195 (31.6)	158 (43.0)	1.20 (0.95 - 1.51), p=0.12
	Vision impairment	167 (30.6)	140 (33.6)	0.85 (0.68 - 1.07), p=0.17
	Hearing impairment	153 (29.1)	96 (26.2)	0.88 (0.64 - 1.20), p=0.40
	Urinary incontinence in past year	127 (22.5)	61 (18.6)	0.80 (0.54 - 1.21), p=0.29
	ADL impairment[d]	137 (25.0)	100 (29.4)	1.01 (0.74 - 1.37), p=0.96
	IADL impairment[e]	106 (20.6)	90 (24.2)	0.98 (0.67 - 1.42), p=0.90
Chronic health conditions	Fair or poor self-rated health	195 (34.2)	160 (43.1)	0.99 (0.80 - 1.24), p=0.95
	High blood pressure	347 (58.9)	234 (57.9)	0.93 (0.79 - 1.11), p=0.43
	Diabetes	152 (25.4)	101 (21.8)	0.79 (0.62 - 1.01), p=0.06
	Chronic lung disease	91 (15.0)	58 (15.4)	1.06 (0.75 - 1.50), p=0.74
	Heart disease[f]	136 (22.5)	97 (25.0)	1.10 (0.80 - 1.51), p=0.54
	Stroke	46 (7.0)	38 (9.7)	1.35 (0.79 - 2.30), p=0.27
	Mental health condition	146 (27.8)	109 (33.8)	1.20 (0.90 - 1.61), p=0.89
	Heavy alcohol use[g]	48 (10.2)	34 (11.7)	0.92 (0.50 - 1.69), p=0.78

Abbreviations: ADL, activity of daily living; IADL, instrumental activity of daily living; RR, relative risk.

[a] The 30 participants who reported they were incarcerated for an unknown duration were excluded from this analysis.

[b] Absolute numbers with weighted percentages are shown.

[c] Adjusted for age, sex, race and ethnicity, wealth, educational attainment, and uninsured status.

[d] Difficulty with bathing, dressing, feeding, toileting, or transferring.

[e] Difficulty with meal preparation, grocery shopping, taking medications, making phone calls, or managing money.

[f] Includes history of myocardial infarction, coronary heart disease, angina, or congestive heart failure.

[g] Defined as more than 4 drinks per day.

© 2023 Garcia-Grossman IR et al. *JAMA Network Open*.
