# Supplementary material for: History of Incarceration and Its Association With Geriatric and Chronic Health Outcomes in Older Adulthood
Source: JAMA Netw Open. 2023 Jan 6;6(1):e2249785. doi: 10.1001/jamanetworkopen.2022.49785 (PMC9856648; doi:10.1001/jamanetworkopen.2022.49785)
Supplement: Supplement 2. — Data Sharing Statement [file jamanetwopen-e2249785-s002.pdf]

Data Sharing Statement

Garcia-Grossman. History of Incarceration and Its Association With Geriatric and Chronic Health Outcomes in Older Adulthood. *JAMA Netw Open*. Published January 06, 2023. doi:10.1001/jamanetworkopen.2022.49785

Data
Data available: No

Additional Information
Explanation for why data not available: The data are already publicly available via the Health and Retirement Study.
